# Successive Deprotonation Steering the Structural Evolution of Supramolecular Assemblies on Ag(111)

**DOI:** 10.3390/molecules27123876

**Published:** 2022-06-16

**Authors:** Jiwei Shi, Zhanbo Li, Tao Lin, Ziliang Shi

**Affiliations:** 1 Center for Soft Condensed Matter Physics and Interdisciplinary Research, School of Physical Science and Technology, Soochow University, Suzhou 215006, China; 20194208053@stu.suda.edu.cn; 2 College of New Materials and New Energies, Shenzhen Technology University, Shenzhen 518118, China; 2070413008@stumail.sztu.edu.cn

**Keywords:** deprotonation, carboxylic acids, inductive effect, supramolecular self-assembly, metal surfaces, scanning tunneling microscopy

## Abstract

In this study, we demonstrate the structural evolution of a two-dimensional (2D) supramolecular assembly system, which is steered by the thermally activated deprotonation of the primary organic building blocks on a Ag(111) surface. Scanning tunneling microscopy revealed that a variety of structures, featuring distinct structural, chiral, and intermolecular bonding characters, emerged with the gradual thermal treatments. According to our structural analysis, in combination with density function theory calculations, the structural evolution can be attributed to the successive deprotonation of the organic building blocks due to the inductive effect. Our finding offers a facile strategy towards controlling the supramolecular assembly pathways and provides a comprehensive understanding of the 2D crystal engineering on surfaces.

## 1. Introduction

Supramolecular self-assembly confined on metal surfaces has gained attention in recent decades [1,2,3,4,5,6,7,8,9,10,11,12,13]. Such a bottom-up strategy towards the fabrication of two-dimensional (2D) supramolecular nanostructures promises a variety of potential applications in heterogeneous catalysis and nano electronic devices [14,15,16,17,18]. The rational design of a two-dimensional supramolecular structure can be achieved, through the judicious choice of functional endgroups, the design of organic ligands (symmetry/dimension/size), the manipulation of intermolecular interactions (e.g., van der Waals force, hydrogen bonding, metal-organic coordination), and the control of kinetic and thermodynamic processes [19,20,21]. In particular, understanding how molecules interact mutually in a certain environment is essential to guide the self-assembly pathways.

Carboxylic acids are well-known for their use in directing the engineering of functional nanostructures on metal surfaces. Similar to that in solution, carboxylic acids can form hydrogen-bonded frameworks via intermolecular hydrogen bonding, or metal-organic networks through the coordination bonding built between certain metal centers and carboxylates (resulting from a deprotonation reaction) [22,23,24,25,26,27,28,29,30]. Notice that the charge of carboxylates can be balanced by the underlying metallic substrate, which may give metal-carboxylate coordination motifs different from that in solution. Moreover, these distinct bonding motifs can be manipulated readily through thermal treatments of the metal substrate, which thus allows for the judicious control of assembly pathways and the rational design of on-surface supramolecular (coordination) nanostructures. For example, different degrees of deprotonation can drive a hierarchical growth of supramolecular nanostructures based on carboxylic acids on metal surfaces [24,31].

**Scheme 1:**
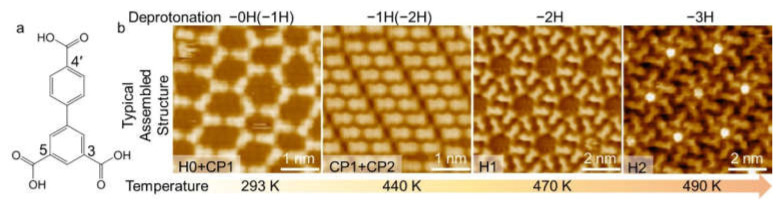
(**a**) Chemical structure of the molecule [1,1′-biphenyl]-3,4′,5-tricarboxylic acid (**BTA**). (**b**) The structural evolution, represented by the typical assemblies obtained after stepwise thermal annealing treatments, depends on the degrees of deprotonation.

In this report, we demonstrate the successive deprotonation of an organic carboxylic acid building block to steer the structural, chiral [31,32,33,34], and bonding motif evolution of a 2D supramolecular assembly system on Ag(111). The molecule ([1,1′-biphenyl]-3,4′,5-tricarboxylic acid, **BTA**) contains three carboxyl endgroups (-COOH) at 3, 4′ and 5 position (Scheme 1a). Due to the inductive effect, the pKa value for the three carboxyl endgroups in aqueous solution increases in order: 3.62, 4.19, and 4.60 [35]. Note that 5-COOH has the highest pKa because the deprotonation of 3-COOH (taking place prior) gives an electron-donating group (3-COO^−^) in the meso position. The increasing pKa values, reflecting the increasing dissociation energies of carboxyl groups, suggest a similar trend in the deprotonation steps for **BTA** on metal surfaces. The Ag(111) surface has an in-between chemical reactivity, with respect to Cu(111) and Au(111), allowing us to resolve all deprotonative states of **BTA** molecules. Scheme 1b depicts the structural evolution represented by the typical structural phases after stepwise thermal annealing treatments for the sample of **BTA** on Ag(111) (see experimental details in Section 4). The structural phase started from (i) a mixture of irregular honeycomb networks and close-packing arrays (H0 + CP1), through (ii) a mixture of close-packing assemblies (CP1 + CP2), and (iii) regular honeycomb networks (H1), to (iv) complex honeycomb networks (H2). Scanning tunneling microscopy (STM) in combination with density function theory (DFT) calculation revealed that the four structural phases emerged in accordance with the increment of the degree of deprotonation (see Scheme 1).

## 2. Results and Discussion

### 2.1. H-Bonded Structures: H0 Networks and Close-Packing Assemblies CP1 and CP2

**Figure 1:**
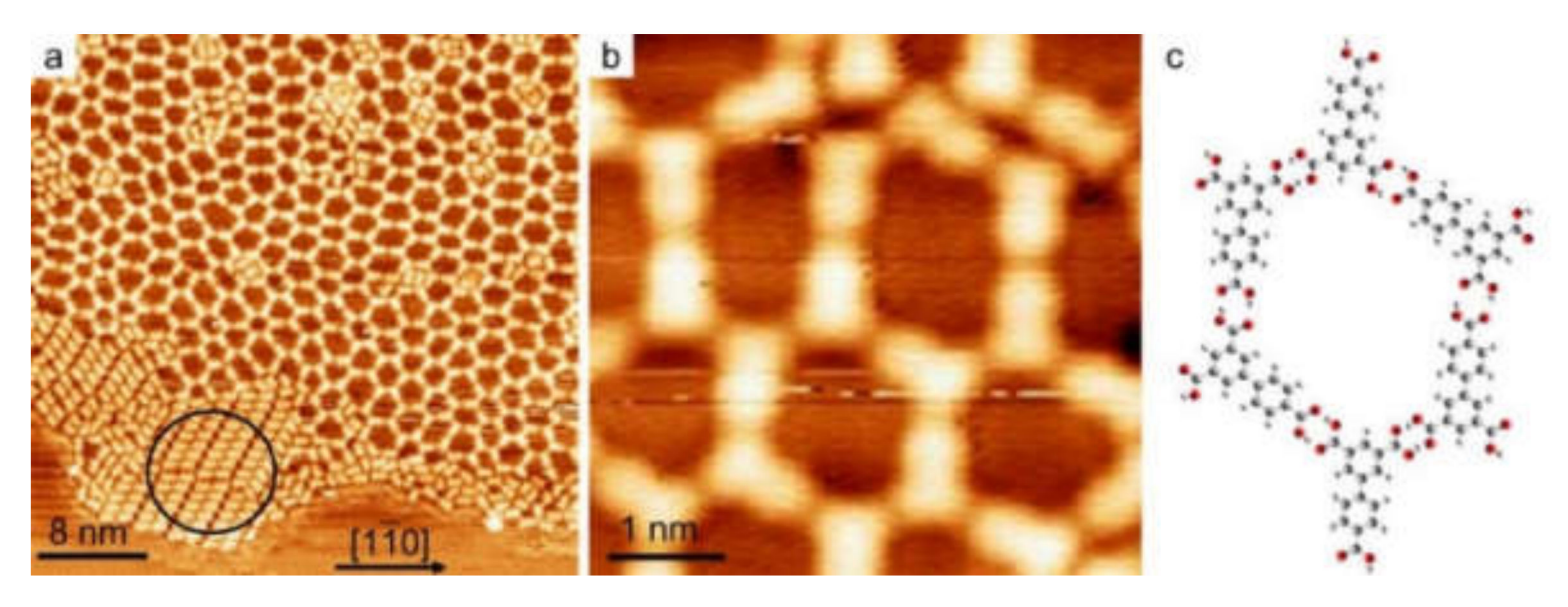
A mixture of H0 networks and CP1 structures at 293 K (room temperature). (**a**) An STM overview (*U* = −1.2 V, *I* = 50 pA). The oval indicates the close-packing structure, CP1. (**b**) The enlarged view of H0 networks. (**c**) A tentative structural model of an H0 cavity. Color: C, grey; O, red; H, white.

Figure 1a depicts a typical STM overview, after the deposition of **BTA** onto a Ag(111) substrate held at 293 K (room temperature (RT)). Irregular honeycomb networks (namely, H0) coexist with close-packing structures (namely, CP1; see the oval in Figure 1a). A close look of H0 networks (Figure 1b) indicates that each hexagonal cavity consists of six molecules, which are aligned with the substrate <1–10> vectors and connected in a corner-to-corner manner. We noted that H0 networks disappeared completely, and the close-packing structures became dominant after the next high-temperature annealing step (*vide infra*). This fact suggests that the **BTA** molecules in H0 remain intact, i.e., none of the carboxyl endgroups undergo the deprotonation reaction. This conclusion can also be supported by the H0 networks obtained on Ag (111) at 120 K and on Au(111) at RT (see Supplementary Materials Figure S1), in which the deprotonation is prohibited because of low temperatures or low chemical reactivity of the surface [26,36]. As illustrated in the tentative structural model for a given cavity (Figure 1c), the molecules are connected by cyclic OH···O bonds; the measured projective distance of H-O is 2.2–2.5 Å. Although this value is slightly smaller than the typical H-bond [37], a longer value is feasible when considering that the carboxyl groups may adopt non-planar configurations.

**Figure 2:**
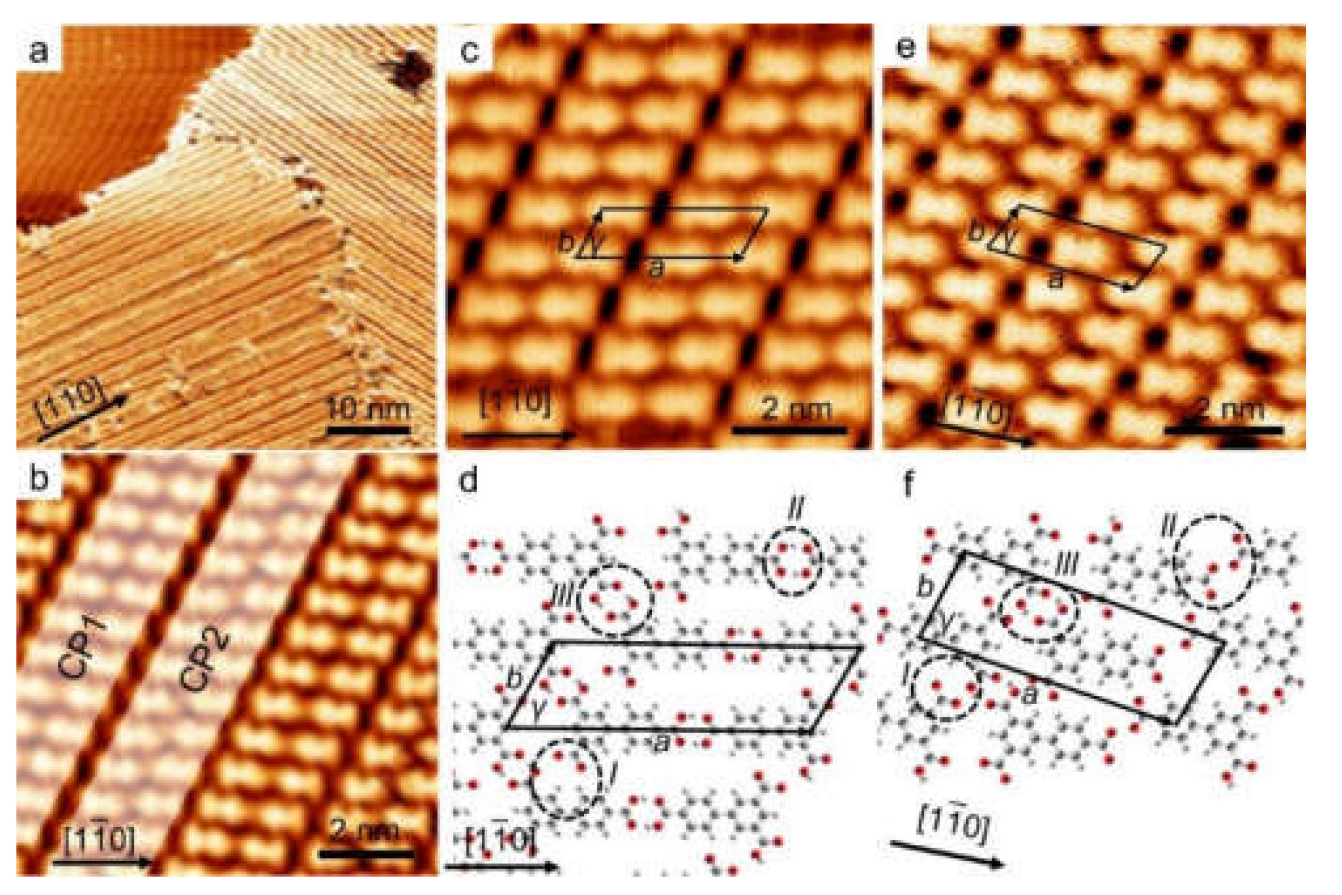
CP1 and CP2 structures after a 440 K annealing. (**a**) An STM overview (*U* = −1.4 V, *I* = 200 pA). (**b**) An STM image (*U* = −0.6 V, *I* = 200 pA) of the domain containing both CP1 and CP2 (highlighted in white). (**c**,**d**) High-resolution STM image (*U* = −1.2 V, *I* = 50 pA) of CP1 and the corresponding structural model. Ovals indicate different intermolecular bonding motifs. (**e**,**f**) High-resolution STM image (*U* = −1.2 V, *I* = 50 pA) of CP2 and the corresponding structural model. Ovals present different intermolecular bonding motifs.

To explore the effect of the degrees of deprotonation on the structural evolution, we annealed the sample to 300–440 K. H0 networks disappeared, while molecular close-packing arrays emerged (Figure 2a). The arrays contain two distinct close-packing structures, namely, CP1 and CP2 (highlighted in white in Figure 2b). In a pure CP1 domain (Figure 2c), every two molecules are connected through a head-to-head link at 4′-COOHs into dimers; the dimers are further packed into arrays. The unit cell of CP1 arrays measures: *a* = 2.90 ± 0.02 nm; *b* = 0.93 ± 0.02 nm; *γ* = 60 ± 2°. These CP1 structures are identified to be the same as the close-packing structure that coexisted with H0 (see the oval in Figure 1a). As CP1 becomes dominant after the thermal annealing treatment, the deprotonation reaction occurs at this stage. A close look at the connections between molecules reveals that both 4′ and 5-COOH are positioned in alignment, while 3-COOH points towards the phenyls of the neighboring molecules (see the unit cell denoted in Figure 2c). According to the pKa values listed above, we propose that one deprotonation occurred at 3-COOH. In our tentative structural model (Figure 2d), we illustrate the interactions between molecules. Ovals *II* and *III* show the intermolecular head-to-head linking via linear O−H···O hydrogen bonds between self-complementary carboxyl endgroups. Oval *I* shows the interactions between the carboxylate (i.e., 3-COO^−^) and phenyl groups that are presumably caused by ionic hydrogen bonds [38]. In addition, our DFT simulation (see calculation details in Supplementary Materials, and Figure S2) using a molecular precursor (namely, **BTA**-1H) with one deprotonation at 3-COOH produces a close-packing assembly, which contains similar intermolecular bonding motifs, and thus supports our tentative model.

As shown in Figure 2e, a pure domain of CP2 differs from CP1 by a slight shift at the otherwise head-to-head links at 4′-COOHs in the latter, while the links at 3 and 5-COOH maintain. The resulting unit cell of CP2 arrays measures *a* = 2.48 ± 0.02 nm, *b* = 0.83 ± 0.02 nm, *γ* = 73 ± 2°. The formation of CP2 is presumably induced by a higher degree of deprotonation. A tentative structural model (Figure 2f) displays the deprotonation occurring at 3 and 4′-COOH (ovals *I* and *II*). In comparison with the neutral hydrogen bond, the strong interaction of the ionic hydrogen bond is the main reason for the shift of head-to-head links between 4′-COOs (oval *II*). The measured interatomic distance (H···O) is ~2.7 Å. The 5-COOH endgroups remain intact (oval *III*) and thus are still linked in a head-to-head manner. This suggests that 5-COOH is of less acidity, because of the pre-deprotonation at 3-COOH, in agreement with the inductive effect [35]. By using a primary precursor with two deprotonations occurring at 3 and 4′-COOH (namely, **BTA**-2H) as the assembly components, our DFT simulation (Supplementary Materials Figure S3) reproduces the major intermolecular bonding motifs, in agreement with our experimental observations. As shown in Scheme 1, in this section, we show two structural phases emerging with the increment of annealing temperatures, which depends on the degrees of deprotonation from −0H(−1H) to −1H(−2H).

### 2.2. H-Bonded Prorous Pattern—H1

**Figure 3:**
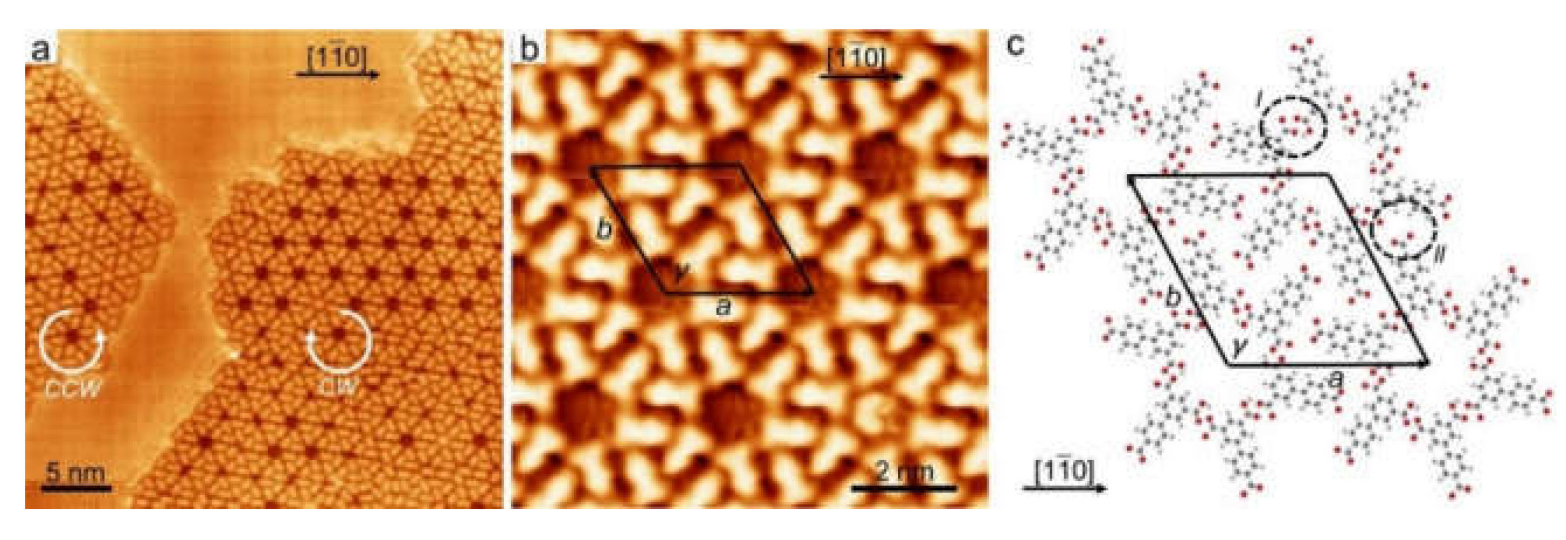
H1 structure after a 470 K annealing. (**a**) An STM overview (*U* = −0.6 V, *I* = 50 pA) of H1 structure containing both *CCW* and *CW* domains. (**b**,**c**) High-resolution STM image (*U* = −0.6 V, *I* = 200 pA) of a *CW* H1 structure and the corresponding tentative structural mode. Ovals indicate the two intermolecular bonding motifs.

After thermal annealing at 470 K, a regular triangular porous pattern (namely, H1) emerged (Figure 3a). In the H1 domain (Figure 3b), each six **BTA** molecules are interconnected roughly in a corner-to-corner manner, and the resulting hexamers are further packed into a domain. The unit cell measures *a* = *b* = 2.86 ± 0.02 nm, *γ* = 120 ± 2°. Interestingly, these hexamers exhibit a chiral feature, and thus two different H1 domains can be discernible on the surface. As shown in Figure 3a, the counter-clockwise (CCW) and clockwise (CW) domains are mirror symmetrical about the substrate <11–2> vectors. Within a given domain, the enantiomeric excess (*ee*) is 100% in terms of the hexamers as the subunits [33,34]. To interpret the formation of these chiral assemblies, we propose a structural model (Figure 3c), in which each molecule in a *CW*-domain undergoes two deprotonations at 3 and 4′-COOH. The primary bonding motifs are marked by the two ovals in Figure 3c. Our DFT simulation (Supplementary Materials Figure S4) using **BTA**-2H molecules as primary building blocks produces a similar chiral pattern, where the proposed intermolecular interactions are feasible. As shown in Scheme 1, different from the former structures, the chirality due to increased degrees of deprotonation emerged at this stage.

### 2.3. Metal-Orgnaic Coordinative Structure—H2 Nework

**Figure 4:**
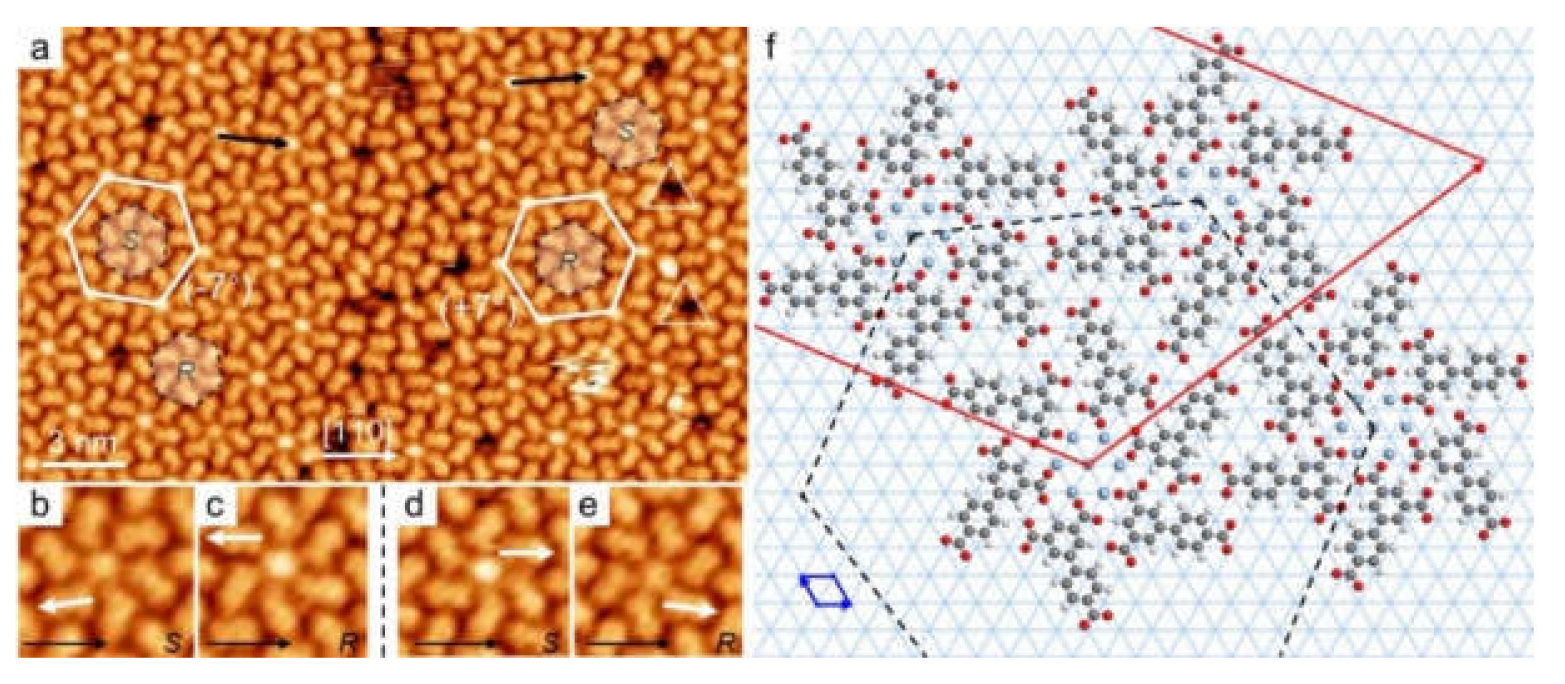
H2 structures after an annealing at 490 K. (**a**) STM overview (*U* = −1.2 V, *I* = 200 pA) of the H2 networks. White hexagons denote the two mirror symmetrical domains (−7°/+7°). Dash hexagons indicate four different **BTA**-hexamers. (**b**–**e**) The four **BTA**-hexamers with different chiral characters, in which white arrows indicate the orientations of **BTA** monomers. The protrusions at the hexamer centers show different contrasts. The black arrows indicate the substrate [1–10] vectors. (**f**) Tentative structural model of a domain (+7°) including four **BTA**-hexamers. Ag_7_ clusters are represented by spheres in cyan. The red lines denote the unit cell of the H2 structure. The blue arrows indicate the substrate unit cell.

After annealing at 490 K, we completed the last deprotonation step. A pure structural phase emerged (Supplementary Materials Figure S5), namely, H2, which consisted of complex honeycomb-patterned domains, and featured distinct chiral and bonding characters. A typical STM overview (Figure 4a) displays two mirror symmetrical domains, which are deviated by ± 7° from the substrate <1–10> vectors, and labeled as domain (+7°) or (−7°). The two white hexagons in Figure 4a define the Wigner–Seitz (W-S) cells corresponding to the two domains and measure a lateral side length of 2.60 ± 0.02 nm. Distinct from H1 structures, a given H2 domain contains two types of **BTA**-hexamers (indicated by the four dash hexagons in Figure 4a) with different chiral characters and orientations. For instance, in domain (+7°), each of the six *S*-hexamers embrace one *R*-hexamer, where the molecules in *S*-hexamers follow the substrate <1–10> vectors, and the ones in *R*-hexamers deviate by −8° from <1–10>. Interestingly, this arrangement leads to an *ee* of 33% for a given domain in terms of the **BTA**-hexamers as subunits. As indicated in the former structures (H0, CP1, and CP2), **BTA** adsorbs on Ag(111) with a preferred orientation along the substrate <1–10> vectors. Thereby, the amplification of chirality can be associated with the favorable adsorption of **BTA** on Ag(111).

The other distinct feature of H2 structures resides in the center of hexamers holding protrusions with two different contrasts. In Figure 4b–e, we show four different types of hexamers from domains (−7°) and (+7°), respectively. Interestingly, the contrast of protrusions shows a relation with the orientation or the position of a hexamer, but not to its chiral character. According to previous reports [39,40], we attribute the protrusion to the accommodation of Ag_7_ clusters. As there exists lattice gas of Ag adatoms coming from the surface step edges or defects on a metal surface [41], we propose that these gas-phase Ag adatoms aggregate into clusters due to a Ag-O coordination bonding with carboxylates. Figure 4f illustrates the two types of **BTA**-hexamers with Ag_7_ clusters inside, in which the Ag_7_ clusters at the six surrounding hexamers are reproduced based on previous DFT calculations [40]. The six outmost Ag atoms interact with carboxylate groups through Ag-O coordination bonds. As shown in Scheme 1, both chiral and coordination bonding characters emerged at this stage.

Based on the above STM analysis and previous theoretical calculations [40], we propose a tentative structural model for the H2 structure with a Ag(111) lattice underneath, in which all molecules are deprotonated because of high temperature annealing. Figure 4f shows the structural model corresponding to a domain (+7°), which has a lattice commensurate with the substrate, giving a matrix notation $\left[\begin{array}{cc} 18 & 11\\ -11 & 7 \end{array}\right]$ with respect to the substrate vectors (the blue arrows in Figure 4f). The W-S cell has a lateral side with a length of 2.62 nm, and an angle deviating by +7° from the substrate [1–10] vector. The cell has six *S*-hexamers positioned on hollow sites and a central *R*-hexamer on the top site. Note that the six *S*-hexamers sit on two different hollow sites (see Figure 4f). This arrangement is also consistent with analysis of the symmetry of a honeycomb lattice, i.e., the surrounding *S*-hexamers represent the C_3_ symmetric points (sitting on hollow sites), while the central *R*-hexamer represents a C_6_ point-group symmetric point (top site).

Our model also rationalizes the contrast difference of Ag_7_ clusters. As illustrated in the model, the Ag_7_ clusters belonging to the surrounding hexamers sit on the hollow sites, in accordance with the previous reports [39,40]. However, at the central hexamer, the Ag_7_ cluster is located at the top site, which may lead to unstable adsorption for the Ag adatoms. This can also result in Ag_7_ clusters collapsing due to fugitive Ag adatoms. Such a scenario agrees with our STM observation that the centers of many central hexamers are dark (see triangles in Figure 4a), i.e., no Ag clusters are present. As the metal-organic coordination motifs can be tuned with distinct coordination numbers and configurations, the incorporation of metal atoms or clusters in supramolecular coordination assemblies may provide new catalytic/electronic functionalities for the resulting nanostructures [8,12]. It is worth further investigating the mechanism of the contrast difference out of the two types of Ag_7_ clusters.

## 3. Conclusions

In this study, we demonstrated that the successive deprotonation of **BTA** molecules steered the structural evolution of the corresponding supramolecular self-assemblies on Ag(111). With the increased degrees of deprotonation, four assembled structures emerged with distinct symmetrical, chiral and bonding characters. Our findings offer a facile strategy to fabricate structurally controlled supramolecular assemblies, and may open new opportunities for the 2D crystal engineering based on supramolecular nanostructures.

## 4. Materials and Methods

The molecule **BTA** (Sigma-Aldrich (Burlington, MA, USA), purity ~96%) was evaporated by an organic molecular beam epitaxy (DODECON Nanotechnology GmbH), and the sublimation temperature was 270 °C. Sample preparation was carried out in an ultrahigh vacuum (UHV) system (SPECS GmbH) at a base pressure of ~3.0 × 10^−10^ mbar. The single-crystal Ag(111) substrate (MaTeck, 99.999%) was cleaned by cycles of Ar^+^ ion sputtering at an energy of 900 eV and annealing at 800 K.

All STM experiments were performed using an in situ Aarhus SPM apparatus controlled by Nanonis electronics. Topographic data were acquired in the constant current mode with the bias voltages applied to the sample. After each thermal annealing treatment of the sample, STM measurements were taken when the sample cooled down to 120 K or 298 K (room temperature). All STM images were processed using WSxM [42].

## Data Availability

Not applicable.

## Supplementary Materials

The following supporting information can be downloaded at: https://www.mdpi.com/article/10.3390/molecules27123876/s1, Figure S1: H0 networks obtained after the deposition of **BTA** on Ag(111) held at 120 K, and on Au(111) at 293 K; DFT calculation details; Figure S2: DFT simulation of CP1; Figure S3: DFT simulation of CP2; Figure S4: DFT simulation of H1; Figure S5: STM overview of H2 networks [43,44,45,46].
