# Supplementary material for: Successive Deprotonation Steering the Structural Evolution of Supramolecular Assemblies on Ag(111)
Source: Molecules. 2022 Jun 16;27(12):3876. doi: 10.3390/molecules27123876 (PMC9231327; doi:10.3390/molecules27123876)
Supplement: Supplementary file 1 [file molecules-27-03876-s001.zip › molecules-1764396-supplementary.pdf]

Supporting Information *for*

# Successive Deprotonation Steering the Structural Evolution of Supramolecular Assemblies on Ag(111)

**Jiwei Shi [1,†], Zhanbo Li [2,†], Tao Lin [2,*], and Ziliang Shi [1,*]**

[1] Center for Soft Condensed Matter Physics and Interdisciplinary Research and School of Physical Science and Technology, Soochow University, 215006, Suzhou, China;

[2] College of New Materials and New Energies, Shenzhen Technology University, 518118, Shenzhen, China;

* Correspondence: lintao@sztu.edu.cn (T.L.); phzshi@suda.edu.cn (Z.S.)

† These authors contributed equally to this work.

**Table of Contents**

1) Figure S1: H0 networks obtained after the deposition of **BTA** on Ag(111) held at 120 K, and on Au(111) at 293 K;

2) DFT Calculation Details;

3) Figure S2: DFT simulation of CP1;

4) Figure S3: DFT simulation of CP2;

5) Figure S4: DFT simulation of H1;

6) Figure S5: STM overview of H2 networks.

1) H0 networks obtained after the deposition of **BTA** on Ag(111) held at 120 K and on Au(111) at 293 K.

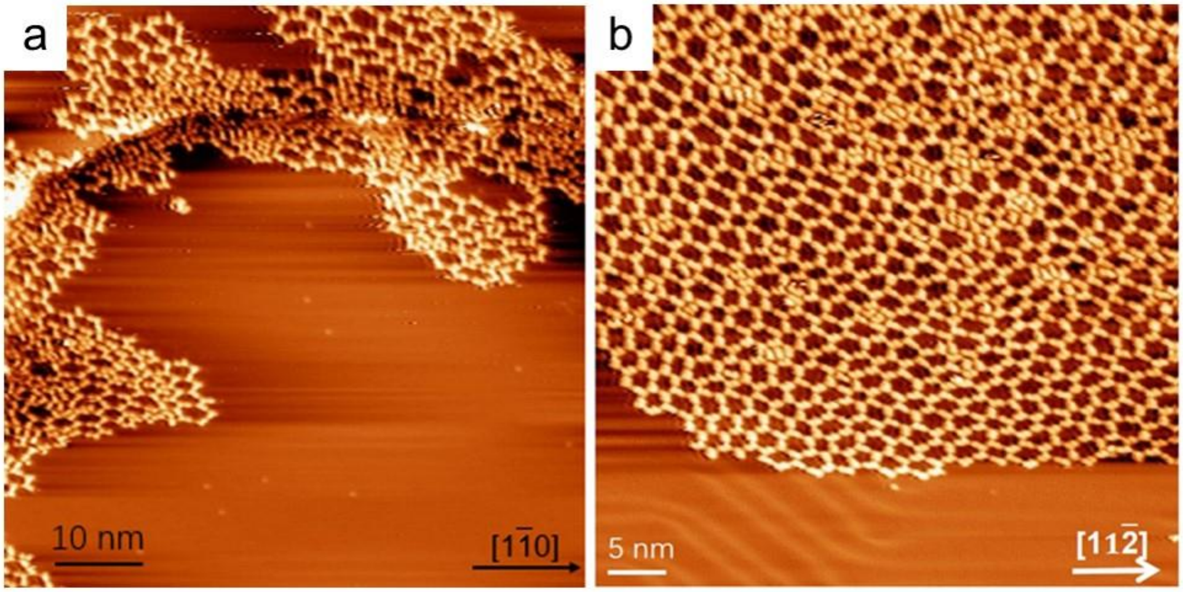


**Figure S1.** (a) STM overview (*U*=-1.2 V, *I*=200 pA, *T*=120 K) of H0 networks obtained after the deposition of **BTA** on Ag(111) held at 120 K. (b) H0 networks on Au(111); *U*=-1.0 V, *I*=100 pA, *T*=293 K.

2) DFT Calculation Details.

Density functional theory (DFT) calculations were carried out using the Vienna Ab initio Simulation Package (VASP)[1] based on the plane-wave basis set. The Perdew-Burke-Ernzerhof (PBE) functional[2] was used to consider the electron exchange and correlation. The ion-electron interactions were described by the projector augmented wave (PAW) method[3]. The DFT-D3 method[4] was used to evaluate the van der Waals (vdW) effect. In all calculations, the kinetic-energy cut off was set to be 400 eV. A vacuum layer was set at least 15 Å and a k-point mesh with 1×1×1 was applied. The bottom Ag layer was kept fixed, while the other atoms were allowed to relax. All structures were fully relaxed until the residue forces acting on all atoms were less than 0.02eV/Å.

3) DFT simulation of CP1.

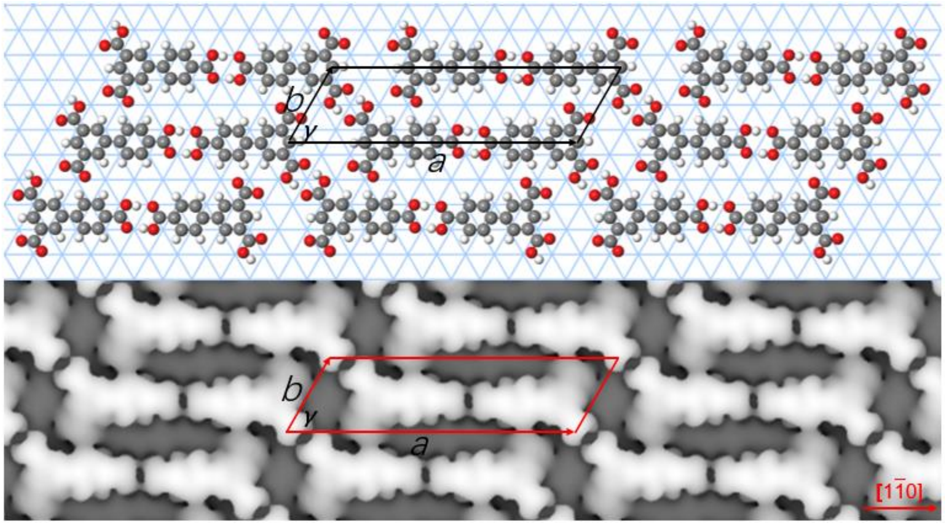


**Figure S2.** DFT calculation corresponding to CP1. $a$=28.7558 Å, $b$=8.6268 Å, $\gamma$=60°.

4) DFT simulation of CP2.

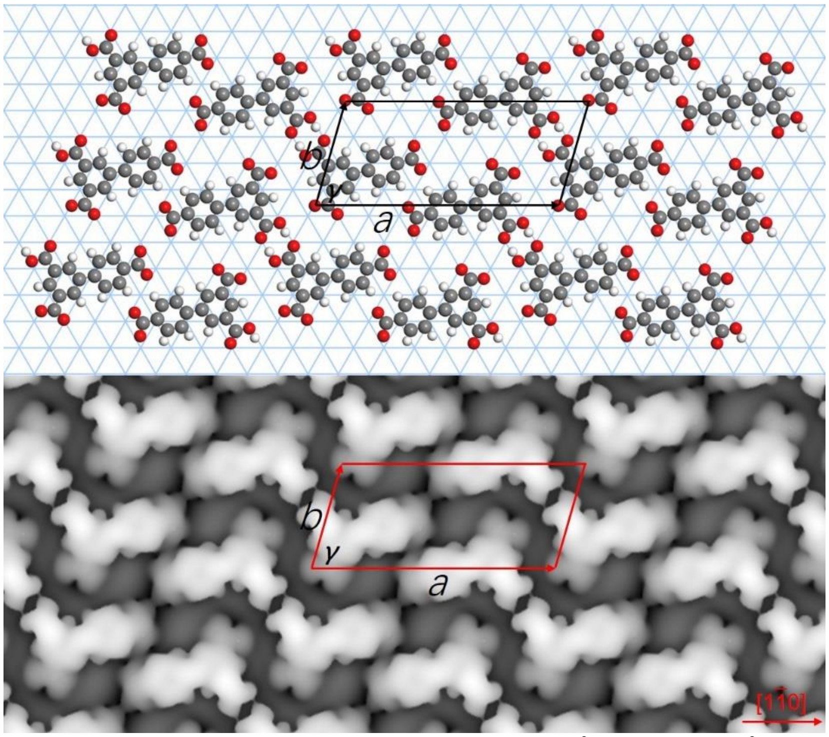


**Figure S3.** DFT calculation corresponding to CP2. $a$=23.0047 Å, $b$=10.3681 Å, $\gamma$=73.8979°.

5) DFT simulation of H1.

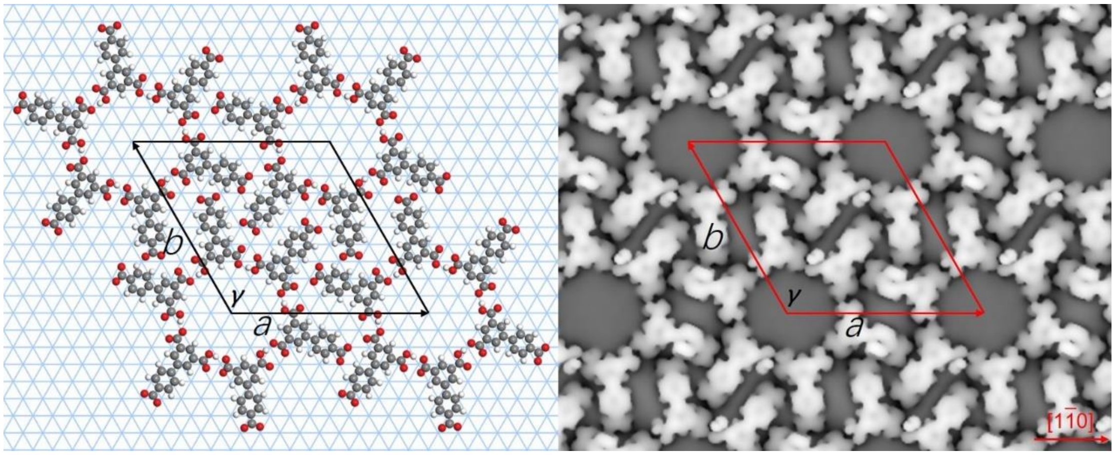


**Figure S4.** DFT calculation corresponding to H1. Unit cell: $a$=28.75585 Å, $b$=28.75585 Å, $\gamma$=120°.

6) STM overview of H2 networks.

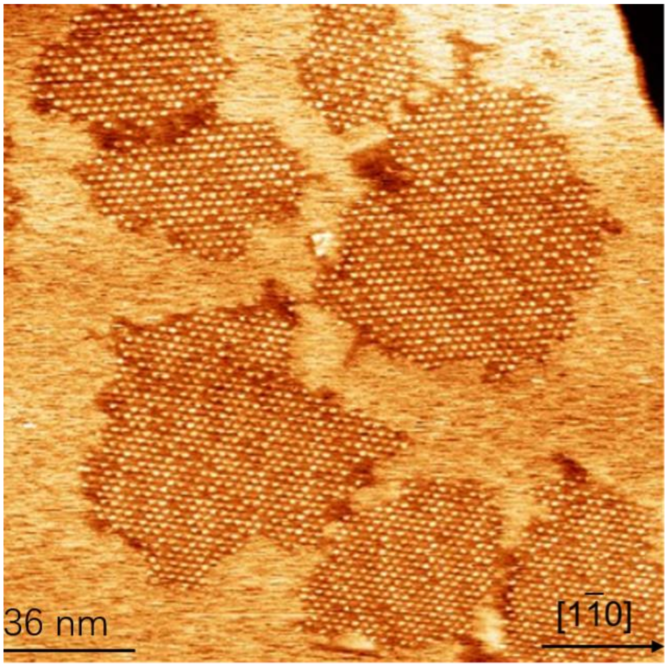


**Figure S5.** Large-scale STM observation ($U$=-1.2 V, $I$=200 pA) of the H2-network domains.
